# Brain Volumetric Measurements in Children With Attention Deficit Hyperactivity Disorder: A Comparative Study Between Synthetic and Conventional Magnetic Resonance Imaging

**DOI:** 10.3389/fnins.2021.711528

**Published:** 2021-10-25

**Authors:** Yingqian Chen, Shu Su, Yan Dai, Zhihua Wen, Long Qian, Hongyu Zhang, Meina Liu, Miao Fan, Jianping Chu, Zhiyun Yang

**Affiliations:** ^1^Department of Radiology, The First Affiliated Hospital, Sun Yat-sen University, Guangzhou, China; ^2^MR Research, GE Healthcare, Beijing, China; ^3^Department of Pediatrics, The First Affiliated Hospital, Sun Yat-sen University, Guangzhou, China

**Keywords:** attention deficit hyperactivity disorder, synthetic MR, brain volumetric measures, gray matter (GM), white matter (WM)

## Abstract

**Objective:** To investigate the profiles of brain volumetric measurements in children with attention deficit hyperactivity disorder (ADHD), and the consistency of these brain volumetric measurements derived from the synthetic and conventional T1 weighted MRI (SyMRI and cT1w MRI).

**Methods:** Brain SyMRI and cT1w images were prospectively collected for 38 pediatric patients with ADHD and 38 healthy children (HC) with an age range of 6–14 years. The gray matter volume (GMV), white matter volume (WMV), cerebrospinal fluid (CSF), non-WM/GM/CSF (NoN), myelin, myelin fraction (MYF), brain parenchyma volume (BPV), and intracranial volume (ICV) were automatically estimated from SyMRI data, and the four matching measurements (GMV, WMV, BPV, ICV) were extracted from cT1w images. The group differences of brain volumetric measurements were performed, respectively, using analysis of covariance. Pearson correlation analysis and interclass correlation coefficient (ICC) were applied to evaluate the association between synthetic and cT1w MRI-derived measurements.

**Results:** As for the brain volumetric measurements extracted from SyMRI, significantly decreased GMV, WMV, BPV, and increased NON volume (*p* < 0.05) were found in the ADHD group compared with HC; No group differences were found in ICV, CSF, myelin volume and MYF (*p* > 0.05). With regard to GMV, WMV, BPV, and ICV estimated from cT1w images, the group differences between ADHD and HC were consistent with the results estimated from SyMRI. And these four measurements showed noticeable correlation between the two approaches (*r* = 0.692, 0.643, 0.898, 0.789, respectively, *p* < 0.001; ICC values are 0.809, 0.782, 0.946, 0.873, respectively).

**Conclusion:** Our study demonstrated a global brain development disability, but normal whole-brain myelination in children with ADHD. Moreover, our results demonstrated the high consistency of brain volumetric indices between synthetic and cT1w MRI in children, which indicates the high reliability of SyMRI in the child-brain volumetric analysis.

## KEY POINTS

- Children with attention deficit hyperactivity disorder show global brain development disability, but normal whole-brain myelination.
- The synthetic MR showed high consistency with conventional T1 weighted images in brain segmentation of children over 5 years.

## Introduction

Attention deficit hyperactivity disorder (ADHD) is the most common childhood-onset neurodevelopmental disorder that may continue through adolescence and adulthood (Biederman and Faraone, 2005), with a prevalence of 1.4–3.0%. Studies of brain volumetric analysis in ADHD have demonstrated its development abnormalities (Valera et al., 2007; Gehricke et al., 2017; Gui et al., 2019). However, the developmental mechanism underlying these changes is still unclear. According to previous studies, the reduction of total brain parenchymal volume (BPV) and gray matter volume (GMV) are consistent findings in individuals with ADHD. With regard to regional differences, the volume reduction predominantly exists in some brain nuclei like the accumbens, amygdala, caudate, hippocampus, and putamen (Hoogman et al., 2017). Specifically, the smaller hippocampal volume was found to correlate with IQ in ADHD patients (Boedhoe et al., 2020). But the change of total white matter volume (WMV) and intracranial volume (ICV) remains controversial (Valera et al., 2007; Frodl and Skokauskas, 2012). Furthermore, studies focused on brain volumetric analysis in children with ADHD rarely characterize the myelin volume, which plays an important role in child development (Paus et al., 2008). Hence, a new approach that can estimate the brain tissue volume, as well as the myelin volume, may be helpful to answer this question.

The currently developed synthetic magnetic resonance imaging (SyMRI) offers a novel approach for brain segmentation with easy data acquisition and rapid postprocessing (less than 1 min) (Hagiwara et al., 2017). Based on assumption that distinct brain structures can be separated by their own quantitative MR values, T1, T2, and PD values can be applied to segment the brain into white matter, gray matter, and cerebrospinal fluid (CSF) tissue fractions, the fraction outside the above components is considered as non-WM/GM/CSF (NON) (Maekawa et al., 2019). The myelin volume can also be calculated with a similar theory by dividing each acquisition voxel into myelin partial volume, excess parenchymal water partial volume, cellular partial volume, and free water partial volume using the model created by Warntjes et al. (2016). All the segmentation steps are performed automatically using dedicated post-processing software called SynthethicMR (Synthetic MR, Linkoping, Sweden), thereafter, eight brain volumetric measurements are estimated including GMV, WMV, CSF, NoN, Myelin, Myelin fraction (MYF), BPV, and ICV (Hagiwara et al., 2017). The robustness of SyMRI in brain volumetric measurements have been demonstrated in several studies (Granberg et al., 2016; Andica et al., 2018). Moreover, by using this technique, a normative database of intracranial volume and brain parenchymal in children has already been created (McAllister et al., 2017). However, no study has reported the related work in children with ADHD.

In the brain volumetric analysis in children with ADHD, much of the previous studies are based on conventional T1 weighted (cT1W) images using the automatic software, such as FreeSurfer (FreeSurfer, MIT, America), FSL (FMRIB, Oxford, United Kingdom), etc. However, the brain segmentation based on cT1W images has some limitations in clinical research. First, the computational process usually takes minutes to hours, which limits its application for clinical use. Second, it requires high contrast between gray and white matter in order to separate the different structures, thus the segmentation results may be influenced by the image quality, the MRI field strength and sequence settings, and even the developmental degree of the brain (Yaakub et al., 2020). Hence, a more convenient and accurate way for brain segmentation and volume estimation is required.

Based on the above literature, in the current study our goal is to investigate the profiles of brain volumetric measurements in children with ADHD using SyMRI. In addition, to test the robustness of SyMRI in the brains of children, the consistency of brain volumetric indices between synthetic and cT1w MRI have been verified.

## Materials and Methods

### Population

This study was approved by the Institutional Review Board of the First Affiliated Hospital of Sun Yat-sen University (No.[2019]328) and informed consent was obtained from all participants’ guardians. The inclusion criteria are: (1) clinically diagnosed ADHD patients or healthy children without ADHD. (2) Age ranges from 6 to 14 years (in order to diminish the impact on maturational heterogeneity caused by a large age range). The exclusion criteria are: (1) having a history of any other nervous system disease or have any brain lesion found by MRI. (2) Have ever received any therapy that may have an impact on nervous system development. (3) Cannot tolerate the MR scan.

A total of 38 pediatric ADHD patients and 38 healthy pediatric volunteers were enrolled in this study during April 2019 to March 2020, with an average age of 9 years. Both groups were comparable in age, sex, and handedness. The male-to-female ratios of both groups are all around 5:1. The 38 ADHD patients included 18 cases of the inattention subtype and 20 cases of the combined subtype, but no case of the hyperactivity-impulsivity subtype. All of the patients were treatment-naïve, i.e., without any drug therapy or behavioral therapy.

### Clinical Assessment

The patients were diagnosed with ADHD based on the criteria of DSM-IV, as well as the parent and teacher reports on Conners Symptom Questionnaire (Conners CK. Conners 3rd Edition. Toronto: Multi-Health Systems; 2008). Both the parent and teacher ADHD indices ≥ 75th percentile were considered to be ADHD positive.

The healthy volunteers were excluded from the diagnosis of ADHD based on the same reports, with both parent and teacher ADHD indices < 75th percentile. All of the participants received parent face-to-face interviews to confirm diagnostic status.

### Magnetic Resonance Imaging Protocol

Whole-brain MRIs were collected on a GE 3.0 Tesla scanner (SIGNA Pioneer MR, GE Healthcare, America). A 3-dimensional localizer scan was performed for the placement of the scanning area. Then a coronal T2-weighted sequence was collected to rule out any cranial lesion, followed by a sagittal three-dimensional T1-weighted fast -spoiled gradient echo-based sequence (T1w FSPGR) (TR = 8.5 ms, TE = 3.3 ms, flip angle = 12°, thickness = 1 mm/no gap, pixel size = 1.0 mm × 1.0 mm, NEX = 1.00, scanning time = 5.5 min). For SyMRI, a two-dimensional multiple-dynamic multiple-echo (MDME) pulse sequence, comprising four automatically calculated saturation delay times and two echo times, was applied to acquire the axial sections. The parameters are as follows: TR = 10205.0 ms, TE = 11.3 ms, flip angle = 20°, thickness = 2 mm/no gap, pixel size = 2.0 mm × 2.0 mm, NEX = 1.00, ETL = 16, scanning time = 5.5 min.

### Brain Tissue and Myelin Volume

For cT1w images, the segmentation was processed with FreeSurfer (v6.0.0,^1^ Harvard University, Boston, MA, United States) (Fischl, 2012). All scans were analyzed following customary and established methods using the standard recon-all script on FreeSurfer version 6.0.^2^ FreeSurfer output involves several hundred potential volumetric measures. The GMV and ICV were calculated automatically by FreeSurfer. However, the WMV could not be divided directly by the software, which was calculated as the sum of “cerebral WM,” “cerebellar WM,” “brainstem,” and “corpus callosum” (Guo et al., 2019). BPV was assessed by summing the GMV and WMV.

**FIGURE 1:**
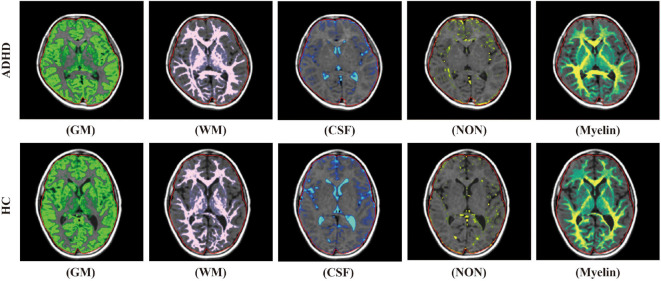
The diagrammatic images of automatic brain segmentation acquired in an ADHD child (upper row) and a child from the control group (lower row) (WM, white matter; GM, gray matter; CSF, cerebrospinal fluid; NON, non-GM/WM/CSF).

For SyMRI, segmentation and volume estimation of myelin and brain tissue, including white matter, gray matter, CSF, and NoN were performed based on the acquired quantitative values, using SyMRI software (version 8.0; Synthetic MR, Sweden). The map of white matter, gray matter, CSF, NoN, as well as the volume of these compositions were automatically calculated according to the process offered by the operation manual. Since SyMRI divided the brain parenchymal into white matter, gray matter, and NoN, BPV was calculated as the sum of the volume of them. ICV was calculated as the sum of BPV and CSF. Brain parenchymal fraction (BPF) was calculated as the ratio of BPV to ICV. Myelin fraction (MYF) was calculated as the ratio of myelin volume to ICV (Figure 1).

### Statistical Analysis

The statistical analysis was performed using the SPSS v21.0 (IBM Corp., Armonk, New York). The Shapiro-Wilk test was used to assess the normality of the data. We compared the demographic data using the chi-square test for all categorical variables and a *t*-test for dimensional data. Pearson correlation analysis and interclass correlation coefficient (ICC) were used to evaluate the association between SyMRI and T1 derived measurements (including GMV, WMV, BPV, and ICV). Analysis of covariance was used to compare the segmented global structural volumes and related fractions measured by both methods separately, controlled for age and gender. A value of *P* < 0.05 was considered significant.

## Results

### Demographic Data

As Table 1 shows, children with ADHD were not significantly different from those children in the control group on the matching variables of age and sex distribution (Table 1).

**TABLE 1:** Demographic data of all patients and control subjects.

	**ADHD (*n* = 38)**	**HC (*n* = 38)**	***p*-value (*t*-test or c^2^)**
Age (y)	8.89 ± 1.89	8.45 ± 1.84	0.378
Male/Female(n)	33/5	32/6	1.00

*ADHD, attention deficit/hyperactivity disorder; HC, healthy control; c^2^, Chi-square test.*

### Comparison of the Synthetic Magnetic Resonance Imaging and Image Conventional T1 Weighted Image Magnetic Resonance Imaging Measurements

**FIGURE 2:**
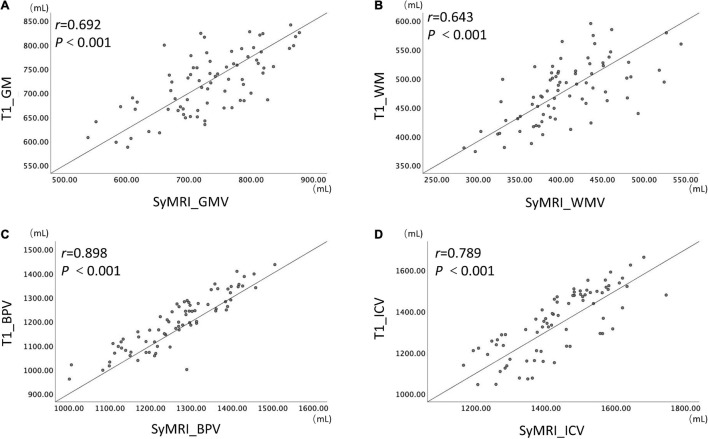
The results of correlation analysis of the SyMRI and cT1w MRI measurements.

The measurements of GMV, WMV, BPV, and ICV all showed noticeable correlation and consistency between the two brain volume estimation methods (*r*-values are 0.692, 0.643, 0.898, 0.789, respectively, *p* < 0.001, ICC values are 0.809, 0.782, 0.946, 0.873, respectively) (Figure 2).

### Comparison of the Specific Brain Tissue Between Attention Deficit Hyperactivity Disorder and Healthy Children Groups With Different Segmentation Methods

**FIGURE 3:**
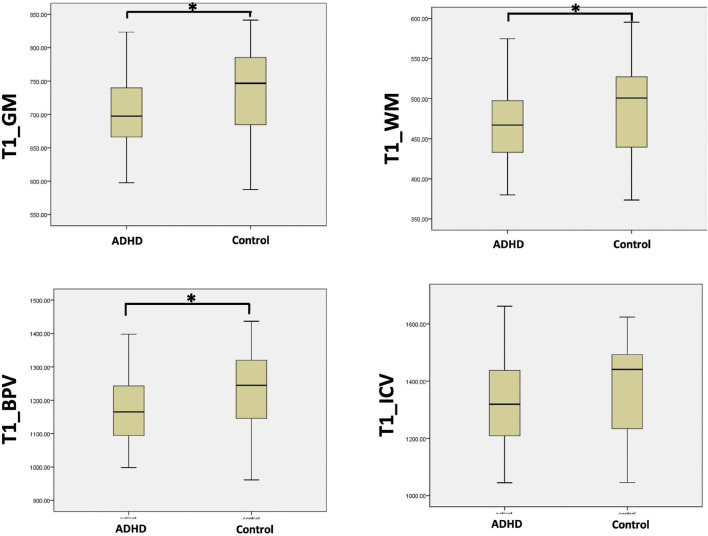
The comparison of the brain tissue volume between ADHD group and control group with the measurements acquired by cT1W images (^∗^*p* < 0.05).

Comparing the brain segmentation volume measured with cT1w images in two groups, GMV (705.8 mL ± 61.7 vs. 734.8 mL ± 63.4, *p* < 0.05), WMV (465.0 mL ± 44.8 vs. 491.0 mL ± 57.4, *p* < 0.05), BPV (1170.8 mL ± 102.0 vs. 1225.8 mL ± 117.0, *p* < 0.05) were significantly smaller in the ADHD group than the control group. While the ICV was comparable in the two groups (1320.2 mL ± 153.7 vs. 1375.3 mL ± 154.6, *p* > 0.05) (Figure 3).

When comparing the specific brain tissue volume acquired by SyMRI within each group, the ADHD group was also shown to have smaller GMV (700.1 mL ± 74.5 vs. 762.8 mL ± 67.5, *p* < 0.01), smaller WM V (388.7 mL ± 47.6 vs. 421.3 mL ± 56.0, *p* < 0.01), smaller BPV (1238.5 mL ± 99.6 vs. 1294.7 mL ± 115.3, *p* < 0.05), and similar ICV (1415.7 mL ± 125.2 vs. 1451.8 mL ± 132.0, *p* > 0.05).

**FIGURE 4:**
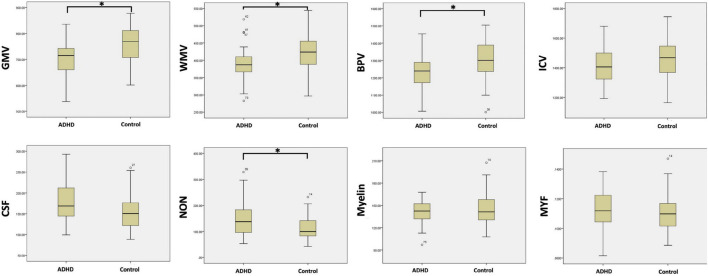
The comparison of the brain tissue and myelin volume between ADHD group and control group with the measurements acquired by SyMRI (^∗^*p* < 0.05).

Compared with cT1w MRI, more parameters can be measured by SyMRI, like CSF volume, NON volume, myelin volume, and myelin fraction. The NON volume of the ADHD group was statistically larger than the control group (164.6 mL ± 66.4 vs. 110.0 mL ± 46.6, *p* < 0.05). While the CSF volume, myelin volume, and myelin fraction of the two groups did not show any statistical difference (177.3 mL ± 44.8 vs. 157.7 mL ± 43.9; 140.3 mL ± 15.7 vs. 143.1 mL ± 23.0; 0.114 ± 0.013 vs. 0.110 ± 0.013, *p* > 0.05) (Figure 4).

## Discussion

In this study, we present the data of the specific brain structure and myelin volumetry in children with ADHD. The reduction of GMV, WMV, BPV, and BPF, and an increase of NON, were found in our study. While the myelin volume, MYF, and ICV were not found to have any statistical significance between the two groups. What is more, we also explore the consistency of brain segmentation with cT1w imaging and SyMRI in pediatric patients with ADHD and healthy children.

### The Brain Structure and Myelin Volume Estimation in Attention Deficit Hyperactivity Disorder

As a kind of developmental disorder, ADHD was thought to correlate with brain development disability, as well as abnormal neural network interplay (Friedman and Rapoport, 2015). The GMV and BPV decrease in pediatric ADHD patients observed in our study were consistent with the previous studies (Valera et al., 2007; Albajara Saenz et al., 2019). Though the WMV change remains controversial, the reduction in WMV observed in our study by using two different segmentation methods also adds evidence to the similar results of previous studies (Valera et al., 2007). Once again, these results demonstrate the global brain development disability of ADHD. However, the regional gray matter or WMV reductions are less conclusive as the reduction regions are not accordant in the results of previous studies (Makris et al., 2015; Francx et al., 2016; Norman et al., 2016). Some studies even failed to find out the brain regions that have between-group differences (Maier et al., 2016; Hoogman et al., 2019). These might suggest that the investigation of microstructure change other than volume change in specific brain regions may be more meaningful for further study. With the use of SyMRI, our study also indicated the NoN volume increased in the pediatric ADHD patients. Although the anatomical basis and physiological implication of the NoN are still unclear, as the diagrammatic images showed in Figure 1, the vessel and perivessel space, the pia mater, some of the choroid plexus and the T2WI-hyperintense white matter add up to form the NoN. In a previous study, enlarged para-vessel spaces were observed in ADHD patients (Vilor-Tejedor et al., 2019), which may partially contribute to the difference. Nevertheless, the specific component and physiological change lead to the difference are still needed to study.

Several imaging techniques have been used in previous studies to assess the integrity of myelin in white matter, such as diffusion tensor imaging, magnetization transfer (MT) imaging, and myelin water imaging. However, the indices estimated from these methods could not specifically reveal the myelin content. Unlike previous studies, our study directly calculated the myelin volume in ADHD by SyMRI methods, which offers a new insight into the myelin maturation of white matter in the developing brain. The results of our study also indicated that though the reduction of WM volume has been demonstrated in children with ADHD, the myelin volume did not show any difference between groups. Considering that most of the myelin is located in white matter, these results may indicate that the maldevelopment of glial cells, other than myelination, contributes to the alteration of white matter microstructure in the drug naïve children with ADHD. Moreover, the altered myelination may only exist in a specific region but not in the whole cerebral white matter, which has been indicated by another study using MT imaging (de Zeeuw et al., 2012). More studies on the specific white matter composition change and white matter change in specific brain regions of ADHD patients are needed to prove this deduction.

### The Comparison Between Synthetic and Conventional Magnetic Resonance Imaging

Among all the parameters that can be measured by both methods, e.g., GMV, WMV, BPV, and ICV, the measurements were all shown to have a good correlation between the T1-derived and SyMRI methods, especially the BPV and ICV. What is more, both of the methods get similar results when comparing ADHD patients and the healthy control. Since the T1-derived brain tissue segmentation and volume estimation by FreeSurfer is widely accepted in neuroscience studies, the SyMRI seems to have comparative reliability both in the measurement and the clinic.

However, it was also found in this study that the volumetric output of gray matter and white matter with the two methods did not show a correlation as high as that in BPV and ICV. It may be caused by the different definitions of the brain component in these two softwares. First, the tissue segmentations from SyMRI and cT1w images were computed with different principles. The tissue segmentations from T1 images were based on the voxel intensity and the universal brain segmentation template using FreeSurfer (Fischl, 2012). While SyMRI computes the tissue ratio in each voxel based on a predefined lookup grid to relate the tissue types to the R1-R2-PD space (West et al., 2012). Second, the SyMRI divides the brain tissue not only into gray matter, white matter, and CSF, but also another component, “NoN” – non-GM/WM/CSF. With the widespread use of the SyMRI, the anatomical basis and physiological implication of NoN will be gradually recognized, which might be helpful for the diagnosis of some diseases. Third, limited by its algorithm and the poor contrast between the gray matter and white matter in the brainstem, FreeSurfer cannot divide the gray matter and white matter in the brainstem. The method of calculation of WMV are various in the previous studies. Similar to the study of Guo et al. (2019), in this study we summing the “cerebral WM,” “cerebellar WM,” “brainstem” and “corpus callosum” FreeSurfer variables as the total WMV, which make its values a little larger than those measured by SyMRI. Thus, the BPV and ICV were the two robust volumetric measurements due to the consistency definition between different brain segmentation methods (Biberacher et al., 2016; Granberg et al., 2016). Fourth, the different resolutions of the two sequences may also contribute to the difference.

The SyMRI overcomes the segmentation of the cT1w images in: (1) the ease-of-use and time-saving of the postprocessing program; (2) more quantitative values can be acquired, e.g., myelin volume. The validity of the brain tissue and myelin volume measurement by SyMRI has been verified by several *in vitro* histological experiments (Warntjes et al., 2016, 2017). A comparative study even indicated that SyMRI showed the most relevant result compared to other techniques for myelin fraction estimation (Saccenti et al., 2020). (3) The T1-weighted images, T2-weighted images, and PD-weighted images can be reconstructed directly by SyMRI without any other sequence (Tanenbaum et al., 2017). However, SyMRI was also found to have some limitations compared with T1-derived segmentation in our study. SyMRI was more sensitive to head motion than the T1w FSPGR sequence. Moreover, for the patient with neurodegenerative disease or the children with incomplete myelination, the type of brain tissue may be misjudged by SyMRI due to the alteration in MR signal. The combined application of SyMRI and FreeSurfer may have the potential to solve this deficiency (Fujita et al., 2019).

There are also some limitations of our study. First, the sample size is relatively small. Second, all of the data were collected in the same model of MRI scanner, thus all scanners used were from the same manufacturer. Studies done with multiple vendors are needed in future studies to investigate the intra-scanner variability (Biberacher et al., 2016). Third, in this study, we chose children over 5 years old for study because the FreeSurfer cannot analyze the children under this age. Further studies are needed to investigate the use of SyMRI in children under 5 years old.

In summary, our study indicated the global brain development disability but normal whole-brain myelination of children with ADHD. This study also demonstrated the high consistency of brain segmentation with cT1w image and SyMRI in children with ADHD. In a way, the SyMRI can replace the cT1W images for brain segmentation and volume estimation in children over 5 years.

## Data Availability Statement

The raw data supporting the conclusions of this article will be made available by the authors, without undue reservation.

## Ethics Statement

The studies involving human participants were reviewed and approved by the Institutional Review Board of The First Affiliated Hospital of Sun Yat-sen University (No.[2019]328). Written informed consent to participate in this study was provided by the participants’ legal guardian/next of kin.

## Author Contributions

YC, SS, and ZY contributed to the conception of the study. YD and ZW performed the experiment. LQ, HZ, and ML contributed significantly to analysis and manuscript preparation. YC, SS and LQ performed the data analyses and wrote the manuscript. MF and JC helped perform the analysis with constructive discussions. All authors contributed to the article and approved the submitted version.

## Conflict of Interest

The authors declare that the research was conducted in the absence of any commercial or financial relationships that could be construed as a potential conflict of interest.

## Publisher’s Note

All claims expressed in this article are solely those of the authors and do not necessarily represent those of their affiliated organizations, or those of the publisher, the editors and the reviewers. Any product that may be evaluated in this article, or claim that may be made by its manufacturer, is not guaranteed or endorsed by the publisher.

## Footnotes

1
http://surfer.nmr.mgh.harvard.edu

2
http://freesurfer.net/fswiki/FreeSurferWiki

## Abbreviations

ADHD: attention deficit hyperactivity disorder:
HC: healthy children
MRI: magnetic resonance imaging
SyMRI: synthetic magnetic resonance imaging
cT1W: image conventional T1 weighted image
GMV: gray matter volume
WMV: white matter volume
BPV: brain parenchymal volume
ICV: intracranial volume
CSF: cerebrospinal fluid
NoN: non-WM/GM/CSF
MYF: myelin fraction
ICC: interclass correlation coefficiency
MT: magnetization transfer.

## References

[B1] Albajara Saenz A. Villemonteix T. Massat I. (2019). Structural and functional neuroimaging in attention-deficit/hyperactivity disorder. *Dev. Med. Child Neurol.* 61 399–405. 10.1111/dmcn.14050 30276811

[B2] Andica C. Hagiwara A. Hori M. Nakazawa M. Goto M. Koshino S. (2018). Automated brain tissue and myelin volumetry based on quantitative MR imaging with various in-plane resolutions. *J. Neuroradiol.* 45 164–168. 10.1016/j.neurad.2017.10.002 29132939

[B3] Biberacher V. Schmidt P. Keshavan A. Boucard C. C. Righart R. Sämann P. (2016). Intra- and interscanner variability of magnetic resonance imaging based volumetry in multiple sclerosis. *Neuroimage* 142 188–197. 10.1016/j.neuroimage.2016.07.035 27431758

[B4] Biederman J. Faraone S. V. (2005). Attention-deficit hyperactivity disorder. *Lancet* 366 237–248. 10.1016/S0140-6736(05)66915-2 16023516

[B5] Boedhoe P. S. W. van Rooij D. Hoogman M. Twisk J. W. R. Schmaal L. Abe Y. (2020). subcortical brain volume, regional cortical thickness, and cortical surface area across disorders: findings from the ENIGMA ADHD, ASD, and OCD working groups. *Am. J. Psychiatry* 177 834–843. 10.1176/appi.ajp.2020.19030331 32539527 PMC8296070

[B6] de Zeeuw P. Mandl R. C. Hulshoff Pol H. E. van Engeland H. Durston S. (2012). Decreased frontostriatal microstructural organization in attention deficit/hyperactivity disorder. *Hum. Brain Mapp.* 33 1941–1951. 10.1002/hbm.21335 21826757 PMC6869977

[B7] Fischl B. (2012). FreeSurfer. *Neuroimage* 62 774–781. 10.1016/j.neuroimage.2012.01.021 22248573 PMC3685476

[B8] Francx W. Llera A. Mennes M. Zwiers M. P. Faraone S. V. Oosterlaan J. (2016). Integrated analysis of gray and white matter alterations in attention-deficit/hyperactivity disorder. *Neuroimage Clin.* 11 357–367. 10.1016/j.nicl.2016.03.005 27298764 PMC4893015

[B9] Friedman L. A. Rapoport J. L. (2015). Brain development in ADHD. *Curr. Opin. Neurobiol.* 30 106–111. 10.1016/j.conb.2014.11.007 25500059

[B10] Frodl T. Skokauskas N. (2012). Meta-analysis of structural MRI studies in children and adults with attention deficit hyperactivity disorder indicates treatment effects. *Acta Psychiatr. Scand.* 125 114–126. 10.1111/j.1600-0447.2011.01786.x 22118249

[B11] Fujita S. Hagiwara A. Hori M. Warntjes M. Kamagata K. Fukunaga I. (2019). 3D quantitative synthetic MRI-derived cortical thickness and subcortical brain volumes: scan-rescan repeatability and comparison with conventional T1 -weighted images. *J. Magn. Reson. Imaging* 50 1834–1842. 10.1002/jmri.26744 30968991 PMC6900192

[B12] Gehricke J. G. Kruggel F. Thampipop T. Alejo S. D. Tatos E. Fallon J. (2017). The brain anatomy of attention-deficit/hyperactivity disorder in young adults - a magnetic resonance imaging study. *PLoS One* 12:e0175433. 10.1371/journal.pone.0175433 28406942 PMC5391018

[B13] Granberg T. Uppman M. Hashim F. Cananau C. Nordin L. E. Shams S. (2016). Clinical feasibility of synthetic MRI in multiple sclerosis: a diagnostic and volumetric validation study. *AJNR Am. J. Neuroradiol.* 37 1023–1029. 10.3174/ajnr.A4665 26797137 PMC7963550

[B14] Gui L. Loukas S. Lazeyras F. Huppi P. S. Meskaldji D. E. Borradori Tolsa C. (2019). Longitudinal study of neonatal brain tissue volumes in preterm infants and their ability to predict neurodevelopmental outcome. *Neuroimage* 185 728–741. 10.1016/j.neuroimage.2018.06.034 29908311

[B15] Guo C. Ferreira D. Fink K. Westman E. Granberg T. (2019). Repeatability and reproducibility of FreeSurfer, FSL-SIENAX and SPM brain volumetric measurements and the effect of lesion filling in multiple sclerosis. *Eur. Radiol.* 29 1355–1364. 10.1007/s00330-018-5710-x 30242503 PMC6510869

[B16] Hagiwara A. Warntjes M. Hori M. Andica C. Nakazawa M. Kumamaru K. K. (2017). SyMRI of the brain: rapid quantification of relaxation rates and proton density, with synthetic MRI, automatic brain segmentation, and myelin measurement. *Invest. Radiol.* 52 647–657. 10.1097/RLI.0000000000000365 28257339 PMC5596834

[B17] Hoogman M. Bralten J. Hibar D. P. Mennes M. Zwiers M. P. Schweren L. S. J. (2017). Subcortical brain volume differences in participants with attention deficit hyperactivity disorder in children and adults: a cross-sectional mega-analysis. *Lancet Psychiatry* 4 310–319. 10.1016/S2215-0366(17)30049-4 28219628 PMC5933934

[B18] Hoogman M. Muetzel R. Guimaraes J. P. Shumskaya E. Mennes M. Zwiers M. P. (2019). Brain imaging of the cortex in ADHD: a coordinated analysis of large-scale clinical and population-based samples. *Am. J. Psychiatry* 176 531–542. 10.1176/appi.ajp.2019.18091033 31014101 PMC6879185

[B19] Maekawa T. Hagiwara A. Hori M. Andica C. Haruyama T. Kuramochi M. (2019). Effect of gadolinium on the estimation of myelin and brain tissue volumes based on quantitative synthetic MRI. *AJNR Am. J. Neuroradiol.* 40 231–237. 10.3174/ajnr.A5921 30591507 PMC7028643

[B20] Maier S. Perlov E. Graf E. Dieter E. Sobanski E. Rump M. (2016). Discrete global but no focal gray matter volume reductions in unmedicated adult patients with attention-deficit/hyperactivity disorder. *Biol. Psychiatry* 80 905–915. 10.1016/j.biopsych.2015.05.012 26115789

[B21] Makris N. Liang L. Biederman J. Valera E. M. Brown A. B. Petty C. (2015). Toward defining the neural substrates of ADHD: a controlled structural mri study in medication-naive adults. *J. Atten. Disord.* 19 944–953. 10.1177/1087054713506041 24189200 PMC4009385

[B22] McAllister A. Leach J. West H. Jones B. Zhang B. Serai S. (2017). Quantitative synthetic MRI in children: normative intracranial tissue segmentation values during development. *AJNR Am. J. Neuroradiol.* 38 2364–2372. 10.3174/ajnr.A5398 28982788 PMC7963732

[B23] Norman L. J. Carlisi C. Lukito S. Hart H. Mataix-Cols D. Radua J. (2016). Structural and functional brain abnormalities in attention-deficit/hyperactivity disorder and obsessive-compulsive disorder: a comparative meta-analysis. *JAMA Psychiatry* 73 815–825. 10.1001/jamapsychiatry.2016.0700 27276220

[B24] Paus T. Keshavan M. Giedd J. N. (2008). Why do many psychiatric disorders emerge during adolescence? *Nat. Rev. Neurosci.* 9 947–957. 10.1038/nrn2513 19002191 PMC2762785

[B25] Saccenti L. Hagiwara A. Andica C. Yokoyama K. Fujita S. Kato S. (2020). Myelin measurement using quantitative magnetic resonance imaging: a correlation study comparing various imaging techniques in patients with multiple sclerosis. *Cells* 9:393. 10.3390/cells9020393 32046340 PMC7072333

[B26] Tanenbaum L. N. Tsiouris A. J. Johnson A. N. Naidich T. P. DeLano M. C. Melhem E. R. (2017). Synthetic MRI for clinical neuroimaging: results of the magnetic resonance image compilation (MAGiC) prospective, multicenter, multireader trial. *AJNR Am. J. Neuroradiol.* 38 1103–1110. 10.3174/ajnr.A5227 28450439 PMC7960099

[B27] Valera E. M. Faraone S. V. Murray K. E. Seidman L. J. (2007). Meta-analysis of structural imaging findings in attention-deficit/hyperactivity disorder. *Biol. Psychiatry* 61 1361–1369. 10.1016/j.biopsych.2006.06.011 16950217

[B28] Vilor-Tejedor N. Alemany S. Forns J. Cáceres A. Murcia M. Macià D. (2019). Assessment of susceptibility risk factors for ADHD in imaging genetic studies. *J. Atten. Disord.* 23 671–681. 10.1177/1087054716664408 27535943

[B29] Warntjes J. B. M. Persson A. Berge J. Zech W. (2017). Myelin Detection using rapid quantitative MR imaging correlated to macroscopically registered luxol fast blue-stained brain specimens. *AJNR Am. J. Neuroradiol.* 38 1096–1102. 10.3174/ajnr.A5168 28428209 PMC7960095

[B30] Warntjes M. Engstrom M. Tisell A. Lundberg P. (2016). Modeling the presence of myelin and edema in the brain based on multi-parametric quantitative MRI. *Front. Neurol.* 7:16. 10.3389/fneur.2016.00016 26925030 PMC4756127

[B31] West J. Warntjes J. B. Lundberg P. (2012). Novel whole brain segmentation and volume estimation using quantitative MRI. *Eur. Radiol.* 22 998–1007. 10.1007/s00330-011-2336-7 22113264

[B32] Yaakub S. N. Heckemann R. A. Keller S. S. McGinnity C. J. Weber B. Hammers A. (2020). On brain atlas choice and automatic segmentation methods: a comparison of MAPER & FreeSurfer using three atlas databases. *Sci. Rep.* 10:2837. 10.1038/s41598-020-57951-6 32071355 PMC7028906

